# Data on a temperature-dependent thermic and electrical properties of a novel blend polymeric system based on poly(vinyl alcohol), chitosan and phosphoric acid

**DOI:** 10.1016/j.dib.2020.105203

**Published:** 2020-01-27

**Authors:** Jonathan Vera, Edgar Mosquera, Jesús Evelio Diosa

**Affiliations:** a Departamento de Física, Universidad del Valle, A.A. 25360, Cali, Colombia; b Centro de Excelencia en Nuevos Materiales (CENM), Universidad del Valle, A.A. 25360, Cali, Colombia

**Keywords:** Polymer electrolytes, Phosphoric Acid, DC conductivities, Thermal properties

## Abstract

In this work, data on a temperature-dependent thermic and electrical properties in a novel blend polymer electrolyte membranes based on poly(vinyl alcohol) (PVA) and chitosan (CS) doped with H_3_PO_4_ at different concentrations were prepared by solution casting method. Their phase behavior and ionic conductivity were studied by DSC, TGA and IS. These membranes exhibit good proton conductivity of the order of 10^−2^ Scm^−1^ at 200 °C and the understanding of the H_3_PO_4_ at different concentrations effect in the polymer electrolyte membranes is crucial for possible applications in fuel cells. The data have not been reported nor discussed in the research paper to be submitting.

**Table:** Specifications Table

Subject area	**Physics, Materials**
More specific subject area	Thermic and electrical properties
Type of data	Tables (1–6), figures (2)
How data was acquired	Differential scanning calorimetry (DSC), thermogravimetric analysis (TGA) and impedance spectroscopy (IS)
Data format	Raw data
Experimental factors	Temperature, heat flow, weight, resistance, conductivity
Experimental features	Very brief experimental description
Data source location	DSC, TGA and IS were recorded in the Laboratory of Phase Transitions in Non-Metallic Systems at Physics Department, Universidad del Valle, Cali, Colombia.
Data accessibility	Data are available within this article
Related research article	This data article is a direct submission to data in brief having as reference the works of Benítez et al. [1] and Quintana et al. [2] on Membranes based on PVA and CS, and Vera et al., to be submitted [3]

**Table:** 

**Value of the Data** • Weight percent loss a different temperature regions give information about the thermal stability which is important to know the work temperature. • Temperature dependent data of heat flow give the characteristic values (glass transition, melting point, decomposition temperature and their enthalpy changes) of the membranes • Temperature dependent data of resistance and conductivity provide a detailed insight to the membranes and their possible application to fuel cells.

## 1 Data

The DC conductivity, σ_0_, can be determined from the resistance of the volume of the sample obtained from the impedance graphs, Nyquist plots (-ImZ vs ReZ), by extrapolating the circular part of the spectrum to the real axis Z′, using σ_0_ = d/AR, where R is the intercept with the Z′ axis, d is the thickness of the membrane and A the contact area of the sample with the electrodes. It is also possible to determine σ_0_ from the adjustment of the experimental data to the Jonscher model [4],

(1) σ′(ω) = σ_0_ + Aω^n^

where σ_0_ is the DC conductivity (independent of the frequency), A is a pre-exponential factor related to the frequency of regime change, ω_p_, as A = σ_0_/(ω_p_)^n^ and n is a value between 0 and 1, where the values of n close to zero indicate that the correlation between the ions is greater than for the values close to 1, which would be the case where the ionic jumps are random (Debye model). From the impedance data, Z ′(ω) and Z″(ω), the values of the real conductivity, σ′ were obtained using the relation,

(2) $$\sigma \prime \left(\omega \right)=\frac{Z\prime \prime }{\left(Z\prime^{2}+Z{\prime \prime }^{2}\right)}$$

The experimental data (TGA, DSC and IS) are reported in Table 1, Table 2, Table 3, Table 4, Table 5, Table 6.

• The Table 1 shows the weight percent loss in three different temperature regions for all the membranes.
• Table 2 shows the characteristic values of the membranes using DSC.
• Table 3, Table 4 show the resistance values of the membranes extrapolated from the Nyquist diagrams in relation to the temperature and concentration of phosphoric acid.
• Table 5 shows the membrane parameters and activation energies for two temperature regions using the Arrhenius model.
• Table 6 shows the parameters obtained from Jonscher model adjustment to the membranes with (PVA:CS) + 10% H_3_PO_4_.

**Table 1:** Weight percent loss in three different temperature regions.

Solution	Weight percent loss in three different temperature regions		
	(30–170)°C	(170–310)°C	(310–450)°C
PVA	4.89%	68.22%	8.82%
CS	10.88%	33.69%	15.94%
(PVA:CS) (80:20)	8.34%	52.13%	21.81%
(PVA:CS)+ 10% H_3_PO_4_	21.69%	14.74%	10.19%
(PVA:CS)+ 20% H_3_PO_4_	9.78%	32.03%	10.01%
(PVA:CS)+ 30% H_3_PO_4_	10.58%	27.74%	9.31%
(PVA:CS)+ 40% H_3_PO_4_	13.31%	23.97%	11.07%

**Table 2:** Characteristic values of the membranes using the DSC.

Solution	T_g_ (°C)	T_m_ (°C)	ΔH (J/g)	T_d_ (°C)	ΔH (J/g)
PVA	67	203	62.43	273	791.30
CS				280	−117.30
(PVA:CS) (80:20)	53	194	38.66	288	532.80
(PVA:CS) + 10% H_3_PO_4_	56	190	67.49	217	300.00
(PVA:CS) + 20% H_3_PO_4_	41	156	25.11	199	113.00
(PVA:CS) + 30% H_3_PO_4_	37	153	13.09	185	83.63
(PVA:CS) + 40% H_3_PO_4_	20	152	57.14	202	12.74

**Table 3:** Resistance values of the membranes extrapolated from the Nyquist diagrams.

T (°C)	T (K)	R (Ω)_80:20_	R (Ω)_10%_	R (Ω) _20%_	R (Ω) _30%_	R (Ω) _40%_
30	303.15		1.04 × 10^6^	7.59 × 10^3^	1.76 × 10^3^	6.12 × 10^1^
40	313.15	1.11 × 10^6^	5.33 × 10^5^	5.08 × 10^3^	1.47 × 10^3^	5.29 × 10^1^
50	323.15	3.40 × 10^5^	2.78 × 10^5^	3.52 × 10^3^	7.93 × 10^2^	3.81 × 10^1^
60	333.15	6.76 × 10^5^	1.62 × 10^5^	2.55 × 10^3^		2.58 × 10^1^
70	343.15	1.20 × 10^5^	8.92 × 10^4^	1.99 × 10^3^	4.54 × 10^2^	1.08 × 10^1^
80	353.15	1.83 × 10^5^	5.79 × 10^4^	1.66 × 10^3^	3.28 × 10^2^	1.37 × 10^1^
90	363.15	8.42 × 10^4^	4.00 × 10^4^	1.37 × 10^3^	2.61 × 10^2^	1.07 × 10^1^
100	373.15	5.01 × 10^4^	2.66 × 10^4^	1.08 × 10^3^	2.01 × 10^2^	8.94 × 10^0^
110	383.15	3.37 × 10^4^	2.03 × 10^4^	9.73 × 10^2^	1.56 × 10^2^	7.57 × 10^0^
120	393.15	2.49 × 10^4^	1.55 × 10^4^	7.84 × 10^2^	1.17 × 10^2^	6.03 × 10^0^
130	403.15	2.49 × 10^4^	1.25 × 10^4^	7.54 × 10^2^	9.54 × 10^1^	4.93 × 10^0^
140	413.15	1.84 × 10^4^	1.02 × 10^4^	6.69 × 10^2^	7.67 × 10^1^	4.16 × 10^0^
150	423.15	1.55 × 10^4^	9.61 × 10^3^	6.17 × 10^2^	6.26 × 10^1^	3.57 × 10^0^
160	433.15	1.30 × 10^4^	7.21 × 10^3^	5.83 × 10^2^	5.08 × 10^1^	2.87 × 10^0^
170	443.15	1.12 × 10^4^		5.36 × 10^2^	4.51 × 10^1^	2.39 × 10^0^
180	453.15	9.31 × 10^3^	3.90 × 10^3^	4.51 × 10^2^	4.09 × 10^1^	1.89 × 10^0^
190	463.15	9.14 × 10^3^	4.30 × 10^3^	4.02 × 10^2^	3.73 × 10^1^	1.62 × 10^0^
200	473.15	7.41 × 10^3^	3.55 × 10^3^	3.47 × 10^2^	3.41 × 10^1^	1.30 × 10^0^

**Table 4:** Conductivity values of the membranes obtained from Table 3 and σ_0_ = d/AR.

T(°C)	T(K)	$\text{σ}{\left(\text{Ωcm}\right)}_{80:20}^{-1}$	$\text{σ}{\left(\text{Ωcm}\right)}_{10\text{\%}}^{-1}$	$\text{σ}{\left(\text{Ωcm}\right)}_{20\text{\%}}^{-1}$	$\text{σ}{\left(\text{Ωcm}\right)}_{30\text{\%}}^{-1}$	$\text{σ}{\left(\text{Ωcm}\right)}_{40\text{\%}}^{-1}$
30	303.15		1.12 × 10^−8^	4.48 × 10^−6^	1.27 × 10^−5^	2.01 × 10^−4^
40	313.15	1.57 × 10^−8^	2.20 × 10^−8^	6.69 × 10^−6^	1.53 × 10^−5^	2.32 × 10^−4^
50	323.15	5.40 × 10^−8^	4.21 × 10^−8^	9.65 × 10^−6^	2.84 × 10^−5^	3.22 × 10^−4^
60	333.15	2.72 × 10^−8^	7.24 × 10^−8^	1.33 × 10^−5^		4.75 × 10^−4^
70	343.15	1.53 × 10^−7^	1.31 × 10^−7^	1.71 × 10^−5^	4.96 × 10^−5^	1.14 × 10^−3^
80	353.15	1.00 × 10^−7^	2.02 × 10^−7^	2.05 × 10^−5^	6.85 × 10^−5^	8.95 × 10^−4^
90	363.15	2.18 × 10^−7^	2.93 × 10^−7^	2.48 × 10^−5^	8.63 × 10^−5^	1.15 × 10^−3^
100	373.15	3.66 × 10^−7^	4.41 × 10^−7^	3.15 × 10^−5^	1.12 × 10^−4^	1.37 × 10^−3^
110	383.15	5.44 × 10^−7^	5.78 × 10^−7^	3.50 × 10^−5^	1.44 × 10^−4^	1.62 × 10^−3^
120	393.15	7.37 × 10^−7^	7.53 × 10^−7^	4.34 × 10^−5^	1.92 × 10^−4^	2.04 × 10^−3^
130	403.15	7.37 × 10^−7^	9.35 × 10^−7^	4.51 × 10^−5^	2.36 × 10^−4^	2.49 × 10^−3^
140	413.15	9.96 × 10^−7^	1.15 × 10^−6^	5.08 × 10^−5^	2.93 × 10^−4^	2.95 × 10^−3^
150	423.15	1.18 × 10^−6^	1.22 × 10^−6^	5.51 × 10^−5^	3.59 × 10^−4^	3.44 × 10^−3^
160	433.15	1.41 × 10^−6^	1.62 × 10^−6^	5.84 × 10^−5^	4.43 × 10^−4^	4.29 × 10^−3^
170	443.15	1.64 × 10^−6^		6.35 × 10^−5^	4.99 × 10^−4^	5.14 × 10^−3^
180	453.15	1.97 × 10^−6^	3.00 × 10^−6^	7.54 × 10^−5^	5.49 × 10^−4^	6.48 × 10^−3^
190	463.15	2.01 × 10^−6^	2.72 × 10^−6^	8.47 × 10^−5^	6.03 × 10^−4^	7.60 × 10^−3^
200	473.15	2.48 × 10^−6^	3.30 × 10^−6^	9.79 × 10^−5^	6.60 × 10^−4^	9.45 × 10^−3^

**Table 5:** Membrane parameters and activation energies for two temperature regions using the Arrhenius model.

Solution	Area (cm^2^)	Thickness (cm)	E_a_ (eV) (30–90)°C	E_a_ (eV) (100–200)°C
PVA				
CS				
(PVA:CS) (80:20)	1.66	0.03	0.63	0.27
(PVA:CS)+ 10% H_3_PO_4_	1.71	0.02	0.24	0.14
(PVA:CS)+ 20% H_3_PO_4_	1.62	0.06	0.13	0.07
(PVA:CS)+ 30% H_3_PO_4_	1.78	0.04	0.14	0.12
(PVA:CS)+ 40% H_3_PO_4_	1.62	0.02	0.13	0.12

**Table 6:** Parameters obtained from Jonscher model adjustment to the membranes with (PVA:CS) + 10% H_3_PO_4_.

T [°C]	*n*	A	σ_0_ [Scm^−1^]
30	0.5655	2.62E-11	1.62E-08
40	0.5316	5.71E-11	3.18E-08
50	0.5616	5.24E-11	6.20E-08
60	0.5406	8.43E-11	1.05E-07
70	0.6119	3.88E-11	1.91E-07
80	0.6602	2.12E-11	2.96E-07
90	0.6469	2.95E-11	4.18E-07
100	0.6293	4.25E-11	6.19E-07
110	0.6555	3.05E-11	8.14E-07
120	0.7031	1.58E-11	1.06E-06
130	0.7519	5.46E-12	1.42E-06
140	0.4840	3.51E-10	1.68E-06
150			
160	1.1209	2.24E-14	2.38E-06
170			
180	0.9956	2.21E-13	3.86E-06
190	0.3864	2.44E-09	3.99E-06
200	0.1956	6.49E-08	4.46E-06

## 2 Experimental design, materials, and methods

Hydrolyzed poly(vinyl alcohol) (PVA, Mw: 31,000–50,000 g/mol), Chitosan (CS) and phosphoric acid (H_3_PO_4_, Mw: 98g/mol) were obtained from Sigma Aldrich, and used as received without any further purification. A solution of acetic acid at 2% by volume of distilled and deionized water was prepared. Then, a solution of PVA and CS was established at the weight ratio of 80:20. Thus, PVA:CS (80:20) and phosphoric acid at concentrations from 10% to 40% was defined in the mixture of acetic acid and distilled and deionized water.

TGA (Q500, TA Instruments) was used to investigate sample weight changes as a function of time and temperature under a N_2_ atmosphere at a flow rate of 50 ml/min. DSC (Q100, TA Instruments) was used to measure the enthalpies, and temperatures of the various thermal events that might occur in the membranes when they are thermally treated. The electrical characterization of the membranes was done by impedance spectroscopy (IS) using a Wayner Kerr impedance analyzer at an excitation signal of 100 mV and 20 Hz–5 MHz frequency range. The dc conductivity, σ, was calculated from the Nyquist plots (-ImZ vs ReZ). The bulk resistance, *R*_bulk_, was obtained from the intercept of the circular arc of the spectra with the real axis, and using the formula σ = *d/AR*, where *d* is the thickness and *A* the contact area of the sample.

### 2.1 Impedance spectroscopy results

**Fig. 1:**
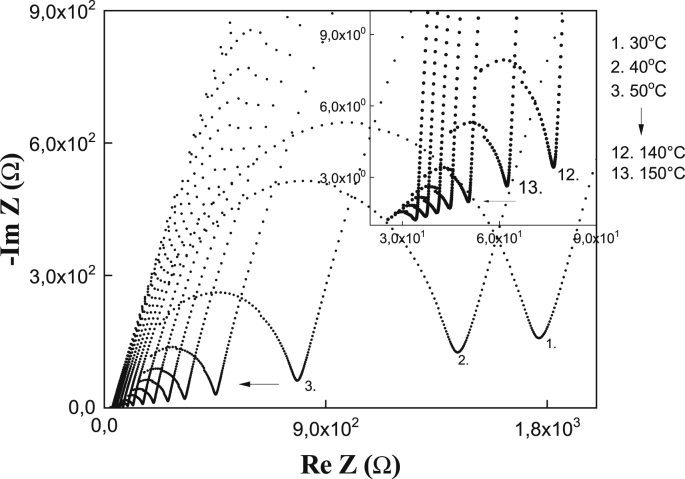
Nyquist diagrams for (PVA:CS) + 30% H_3_PO_4_.

Fig. 1 shows the Nyquist diagrams for (PVA:CS) + 30% H_3_PO_4_ to isotherms between 30 °C and 200 °C, where a semicircle is observed at high frequencies, and which is associated with the electrical response in the volume of the sample. At low frequency regime there is a linear tendency associated with the effects of the interface with the electrodes. The resistance and conductivity values of all membranes is show in Table 3, Table 4

**Fig. 2:**
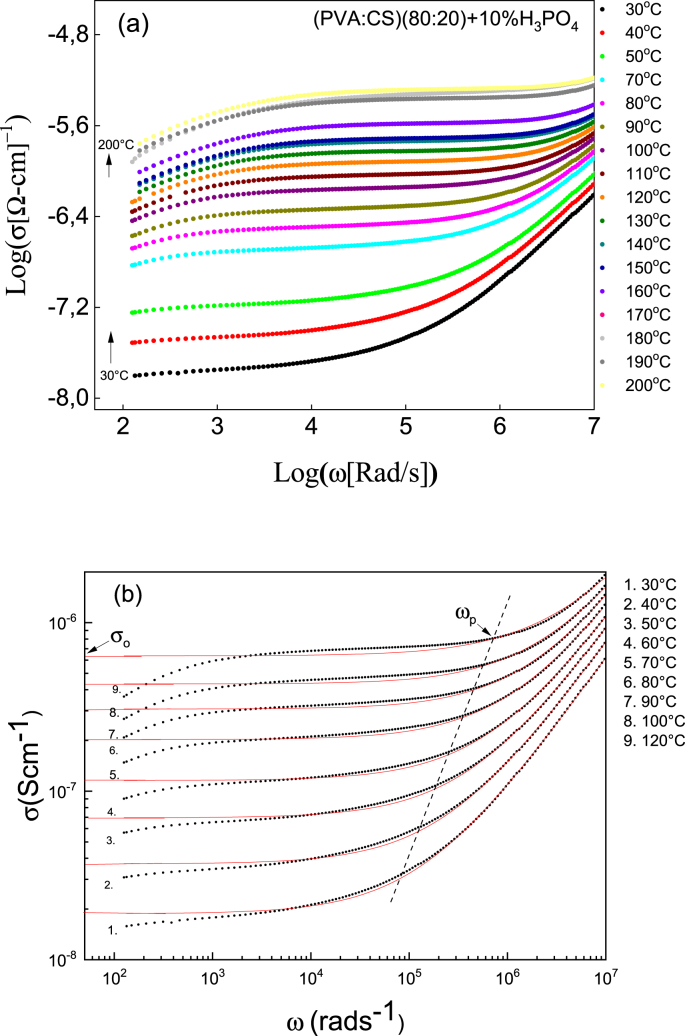
(a) Logarithm of the real part of the AC conductivities as a function of the logarithm of the frequency to different isotherms for (PVA:CS) + 10% H_3_PO_4_. (b) In solid line the fit for typical curves.

Fig. 2a shows the logarithm of the real part of the AC conductivities obtained from ec (2) as a function of the logarithm of the frequency (20 Hz–5 MHz) at several isotherms for (PVA:CS) + 10% H_3_PO_4_. In solid line the fit for typical curves obtained from ec (1) (Fig. 2b) and the parameters are show in Table 6. The DC conductivity (σ_0_) values are in agreement with those calculated from Nyquist plots (see Table 4). On the other hand, the n-exponent parameter, except for 160 °C, takes values between 0 and 1; values greater than 1 could be associated with high values of energy storage in the collective movements of the short-range ions and which cannot be explained by Jonscher model.

## References

[1] Benítez M., Diosa J.E., Vargas R.A. (2018). Effect of H_3_PO_2_ on the mechanical, thermal, and electrical properties of polymers based on poly (vinyl alcohol) (PVA) and chitosan (CS). Ionics.

[2] Quintana D.A., Baca E., Mosquera E., Vargas R.A., Diosa J.E. (2019). Improving the ionic conductivity in nanostructured membranes based on poly(vinyl alcohol) (PVA), chitosan (CS), phosphoric acid (H_3_PO_4_), and niobium oxide (Nb_2_O_5_). Ionics.

[3] J. Vera, E. Mosquera and J. E. Diosa, Temperature-dependent thermic and electrical properties of a novel blend polymeric system based on poly(vinyl alcohol), chitosan and phosphoric acid. to be submitted. 10.1016/j.dib.2020.105203 PMC7005521 32055670

[4] K- Jonscher A. (1996). Universal Relaxation Law.

